# Experimental study on the effect of different cement content on the improvement of dynamic characteristics of seismic-prone poor soil

**DOI:** 10.1371/journal.pone.0300849

**Published:** 2024-05-16

**Authors:** Yunhui Zhao, Feng Qiao, Fanchao Meng, Zhihua Zheng, Jiapei Gu, Haoyu Li

**Affiliations:** 1 College of Geological Engineering, Institute of Disaster Prevention, Langfang, Hebei, China; 2 Hebei Key Lab of Earthquake Disaster Prevention and Risk Assessment, Sanhe, China; China University of Mining and Technology, CHINA

## Abstract

The improvement of sandy soils with poor seismic properties to modify their dynamic characteristics is of great importance in seismic design for engineering sites. In this study, a series of dynamic tests on sandy soils sandy soils with poor seismic conditions were conducted using the GCTS resonant column system to investigate the improvements effects of different cement contents on dynamic characteristic parameters. The research findings are as follows: The cement content has certain influences on the dynamic shear modulus, dynamic shear modulus ratio, the maximum dynamic shear modulus, and the damping ratio of sandy soils with poor seismic properties. Among them, the influence on dynamic shear modulus is limited, while the damping ratio is significantly affected. The addition of cement to seismic-poor sandy soils significantly enhances their dynamic characteristics. The most noticeable improvement is observed when the cement content is 8%. Through curve fitting analysis, a relationship equation is established between the maximum dynamic shear modulus and the cement content, and the relevant parameters are provided. A comparative test between the improved soils and the remolded soils reveals that the addition of cement significantly improves the seismic performance of the poor soils. The recommended values for the range of variation of the dynamic shear modulus ratio and damping ratio are provided, considering the effect of improvement. These research findings provide reference guidelines for seismic design and engineering sites.

## Introduction

China is a country prone to earthquakes. According to data from the China Earthquake Networks Center [1], a total of 2,092 earthquakes with a magnitude of 3.0 or higher occurred in China between 2020 and 2022. Investigations and studies on the damage caused by multiple major earthquakes in China have shown that the properties of geomaterials, especially the dynamic characteristics of soils, have a significant impact on seismic effects [2]. The dynamic characteristics of soils are often represented by two parameters: dynamic shear modulus and damping ratio [3–5]. Research on the rationality and reliability of these two parameters will have a significant influence on the results of seismic response analysis of soil layers. Soils such as sand, which are prone to large deformations and extensive shear failures during earthquakes, can be classified as seismic-poor soils. Currently, there are various types of seismic-poor soils in nature, including conventional soils, such as sandy soils and special soils, such as soft soils. The presence of these seismically poor soils is one of the main causes of severe earthquake damage.

In recent years, many researchers have conducted studies on the modification of poor seismic soils, mainly focusing on the comparison of their dynamic parameters before and after modification. Kostas Senetakis et al. [6–9] investigated the dynamic characteristics of Australian biosand, volcanic soil composed of crushed granite, reconstituted sand with crushed rock particles, and highly porous pumice soil through resonant column tests. The study showed that the shear modulus of the modified sands decreased while the damping ratio increased. Sun Jing et al. [10] studied the variations of dynamic stress, dynamic modulus, modulus ratio, and damping ratio of silt subjected to multiple freeze-thaw cycles under different negative temperatures using dynamic triaxial tests. The results revealed a negative correlation between freeze-thaw cycles and dynamic stress and modulus, and a positive correlation with modulus ratio and damping ratio. Fanchao Meng et al. [11, 12] conducted a series of dynamic characteristic tests on clayey sands using the GCTS resonant column system. The results showed that confining pressure and relative density had an impact on the dynamic shear modulus, the modulus ratio, the damping ratio, and the maximum dynamic shear modulus. Ke Cheng et al. [13] performed resonant column tests on reconstituted silty sands with different amounts of loess content. The results demonstrated that the dynamic shear modulus of the mixture initially decreased and then increased with an increase in loess content, while the damping ratio showed the opposite trend, with a distinct threshold. The threshold values differed between saturated and dry specimens. Baoping Li et al. [14] focused on expansive soils and investigated the variations of dynamic parameters with NaCl solution concentration. The results showed a significant improvement in the dynamic characteristics of the expansive soils after NaCl solution modification. Xu Yongli et al. [15] conducted dynamic triaxial tests to determine the dynamic shear modulus and damping ratio of lime-modified saline soils. The study revealed that the dynamic shear modulus and damping ratio of lime-modified saline soils were influenced by temperature, confining pressure, and frequency, with temperature had a more significant impact on dynamic parameters. Yi Wenni et al. [16] studied the effects of different salt content on the dynamic characteristics of silty soils under cyclic loading using dynamic triaxial tests. The results showed that the salt content had a notable influence on the dynamic modulus and critical dynamic stress of the silty soils, with the dynamic modulus gradually decreasing with increasing salt content. In recent years, domestic and foreign scholars [17–20] conducted dynamic characteristic tests on clay and sandy soils mixed with different sizes of tire particles, investigating the effects of tire size, tire content, and confining pressure on the dynamic characteristics of the soil. The experimental results showed that using waste tire fragments mixed with clay or sand as roadbed fill material was feasible, and that the mixed soil exhibited good seismic energy dissipation performance. Bahar’s [21] article reports on the Algerian experience on earth construction in housing and gives an extended review of an experimental study to investigate a stabilised soil by either mechanical means such as compaction and vibration or chemical stabilisation by cement. Mechanical stabilisation by dynamic compaction seems to give better results as compared to static or vibro-static compaction. A better compressive strength at the dry state and after 48h of immersion in water was obtained with chemical stabilisation at cement content higher than 8%. Du et al. [22] studied the cyclic triaxial behavior of cement-stabilized Organic matter–disseminated sand under various confining pressures, curing periods, and cement contents. Through data fittings, the maximum elastic modulus (E_max_), normalized elastic modulus (E_d_/E_max_), and maximum dynamic damping ratio (λ_max_) were obtained. Then, the effects of cement content, curing time, and initial confining pressure on Emax, E_d_/E_max_ and λ_max_ were systematically analyzed. Finally, the effectiveness of cement stabilization on the dynamic behavior of cement-stabilized Organic matter–disseminated sand was discussed by comparing the data with that of un-cemented Organic matter–disseminated sand. Dai et al. [23] conducted a series of consolidated undrained cyclic triaxial shear tests on cement-stabilized soil with and without super-absorbent-polymer(SAP), and the effects of the effective stress, cyclic stress ratio (CSR), and cyclic number of cycles on the dynamic behaviors were studied. The results show that the SAP can increase the dynamic elastic modulus (approx 10.5%) and damping ratio (approx 11.2%) of cement-stabilized soil, and improve the brittle failure and negative impact on superstructures of cement-stabilized soil under cyclic loading. The influence of CSR on the dynamic elastic modulus and damping ratio of cement-stabilized soil with SAPs has thresholds, and relevant empirical formulas are established.

In summary, current research on the improvement of poor seismic sands focuses on two aspects mainly. First, there are experimental studies on poor seismic soil itself or mixed soils, including the influences of soil physical and mechanical properties, confining pressure, load frequency, etc., on the dynamic shear modulus and damping ratio. Second, there are studies on the dynamic characteristics of poor seismic soils after the addition of materials, with the focus on soft materials such as tires or lime. These studies compare the differences in the dynamic characteristics of poor seismic soils before and after modification. Cement, as the most common and cost-effective material in construction [24], has received little attention in terms of its effectiveness in improving poor seismic soils. Additionally, due to the loose nature of sandy soils, the sampling and disturbance issues are recognized challenges in the geotechnical field, making the research on these problems complex. Existing research results are primarily qualitative analyses, providing improvement effects of materials added to the soil without detailed data analysis. There is a lack of information regarding the effectiveness of different cement contents, such as the increase in dynamic shear modulus when cement is added. In view of this situation, this study conducted experiments to investigate the effects of different cement content on the dynamic characteristics of poor seismic sands. On the contrary, this study engages in quantitative analysis, offering recommended values for the increase in dynamic shear modulus and the decrease in damping ratio resulting from the addition of cement to improve seismically unfavorable sandy soil. In practical engineering applications, engineers can use these recommended values as references by multiplying them with specific soil parameters in their projects. The methods and findings of this study can provide a basis for soil improvement and engineering applications in areas with poor seismic soils.

## Experimental overview

### Experimental equipment

The experimental study used the TSH-100 Resonant Column Apparatus, which was imported from GCTS (Geotechnical Centrifuge Testing Systems), a company based in the United States. This instrument was specifically designed for the study of geotechnical engineering in the School of Geological Engineering of the Institute of Disaster Prevention Science and Technology.

**Fig 1:**
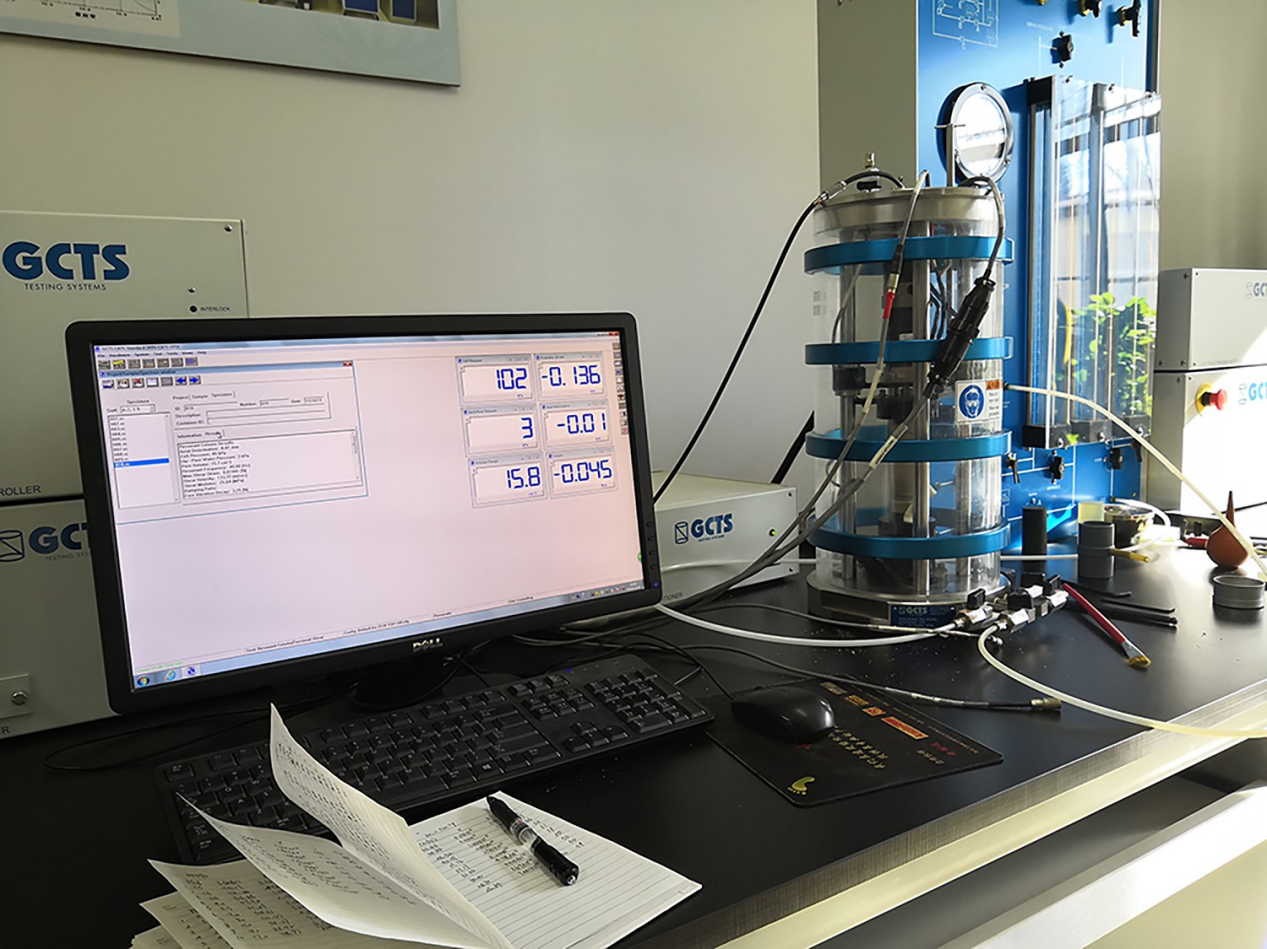
Resonant column tester equipment.

The TSH-100 Resonant Column Apparatus features a floating drive system and an automatic operation system. It has a floating excitation frequency range of 0 to 250 Hz and a shear strain range of 1E-6 to 1E-4. The maximum shear displacement is ±25°, and it can apply a continuous pressure up to 1000 kPa. The general appearance of the equipment is shown in Fig 1.

### Experimental materials

**Fig 2:**
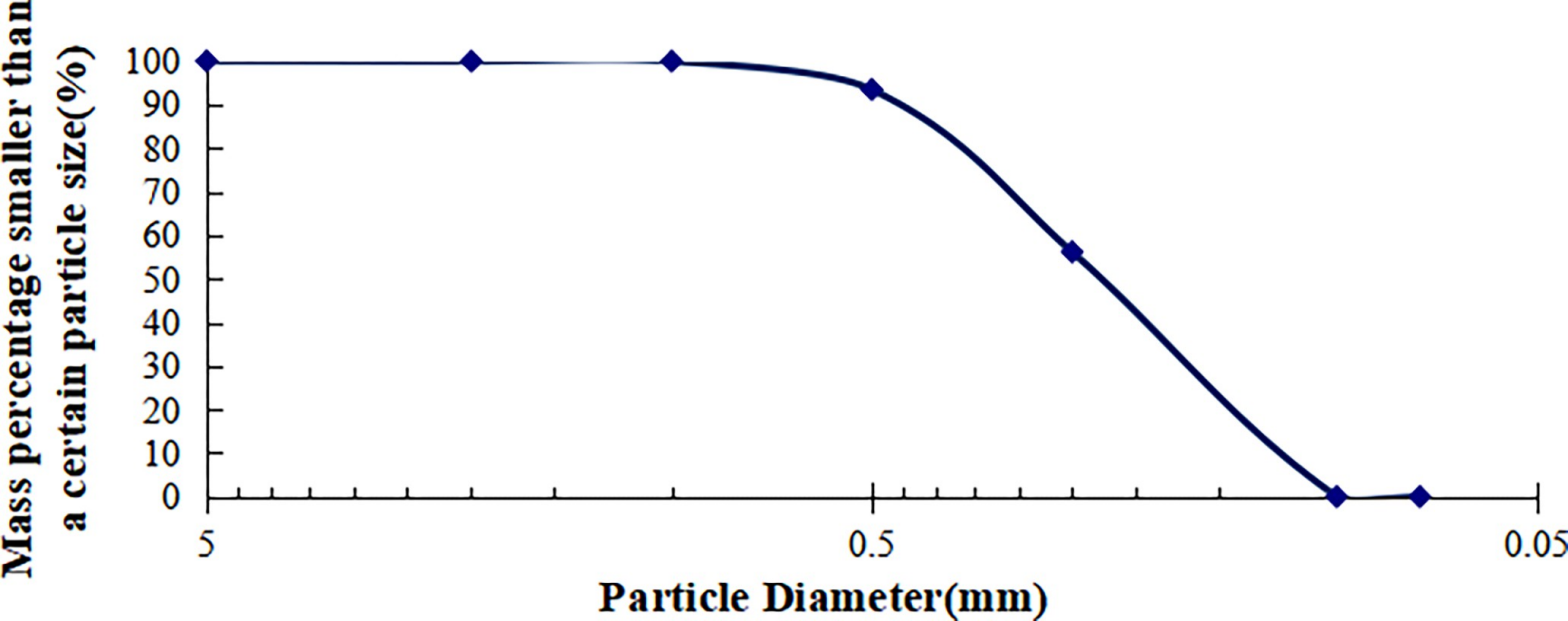
Particle size distribution curve of the test sand (d_max_<1mm).

To facilitate comparison, the experiments used artificially prepared poor seismic soil by adding cement and sugar to standard sand from Fujian. The basic physical properties of the standard sand are presented in Table 1. During sample preparation, particle size distribution tests were conducted on the standard sand (d_max_ < 1mm), and the particle size distribution curve was obtained as shown in Fig 2. The cement added to the sand has a grade of P.O42.5. Sugar dissolves upon contact with water and creating pores, an appropriate amount of sugar was added during sample preparation to simulate the porosity of the soil.

**Table 1:** Basic physical properties of the test sand.

Soil sample	ρ_dmax_	ρ_dmin_	d_60_	C_u_	C_c_	grading
**Standard sand**	1.547	1.344	0.23	2	0.86	badness

### Experimental procedure

Based on preliminary tests and existing research results, when the cement content exceeds 9%, the samples essentially become "concrete" blocks, and the GCTS resonant column apparatus cannot induce vibration in the samples. Therefore, the cement content in this experiment is divided into four levels: 0% (pure sand), 2%, 4%, and 8%. Four consolidation pressures, namely 50 kPa, 100 kPa, 200 kPa, and 300 kPa, were selected to simulate the burial depth of the soil. Furthermore, four reconstituted soil tests were designed under the condition of 4% cement content. In total, 20 independent tests were conducted under different conditions. Considering that sandy soil can be classified into loose, medium-dense, and dense states, this study focused on the most common medium-dense state, selecting a relative density of 50% for the specimens. The specific experimental plan is shown in Table 2.

**Table 2:** Experimental plan.

group	Cement content(%)	Consolidation confining pressure(kPa)	consolidation ratio
**1**	0	50.100.200.300	1
**2**	2	50.100.200.300	1
**3**	4	50.100.200.300	1
**4**	8	50.100.200.300	1
**C-3**	4- remodeling	50.100.200.300	1

### Sample preparation

**Fig 3:**
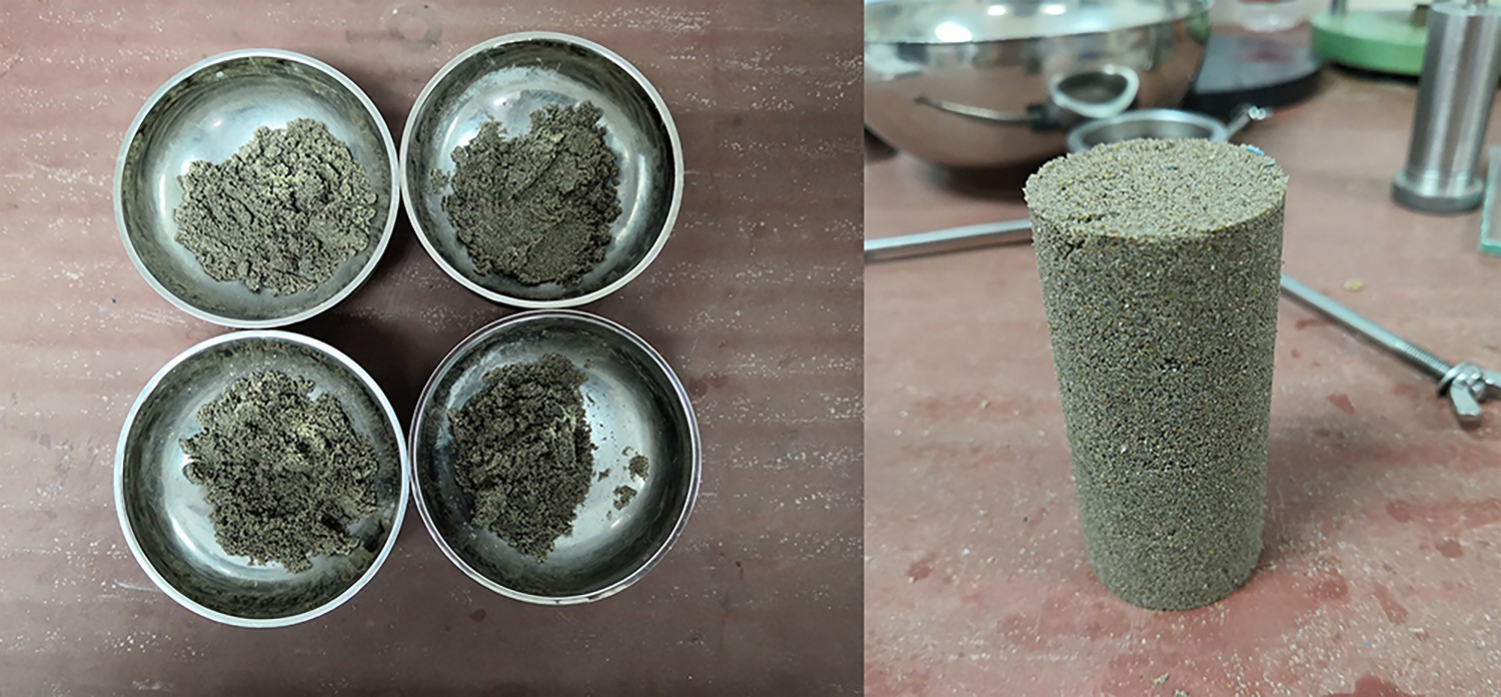
Sample preparation.

The samples were cylindrical with a diameter of 38.1mm and a height of 80mm. The mass of sand and cement required for each sample under each condition set by the test plan was calculated. Then, the calculated sand and cement were weighed into four portions, mixed uniformly, and added to distilled water. To avoid the influence of moisture content on the results, the initial moisture content of all samples was set to 5%. The four portions of soil were packed into a compaction mold with lubricated sidewalls in four layers and compacted to a height of 80mm. After compaction, the sample was removed. The production of the sample is shown in Fig 3.

## 3. The basic formula for calculating dynamic shear modulus and damping ratio in resonant column test

The dynamic shear modulus during torsional resonance can be calculated using the following formula [25]:

$$G_{d}={\left(\frac{2\pi f_{n}h_{c}}{\beta_{s}}\right)}^{2}\rho_{0}$$ (1)

Where *G*_*d*_ is the dynamic shear modulus (MPa), *f*_*n*_ is the measured resonance frequency (Hz), *h*_*c*_ is the height of the consolidated soil specimen(cm), *ρ*_0_ is the density of the specimen (*g*/*cm*^3^), and *β*_*s*_ is the correction factor for frequency and height.

The relationship between dynamic stress and strain in the soil is described as follows [26]:

$$\tau_{d}=\frac{\gamma_{d}}{a+b\gamma_{d}}$$ (2)

From this, the dynamic shear modulus can be obtained.

Simultaneously, the normalized dimensionless expressions can be obtained:

$$\frac{G_{d}}{G_{d\max}}=\frac{1}{1+\gamma_{d}/\gamma_{r}}$$ (3)

In the above equations, *τ*_*d*_ is the dynamic shear stress, *γ*_*d*_ is the dynamic shear strain, *γ*_*r*_ = **a**/*b* is the reference shear strain, and a, b is the experimental parameter determined from the test data. Typically, 1/*b* = *τ*_*ult*_ is referred to as the ultimate shear strength. 1/*a* = *G*_*d*max_ Referred to as the maximum dynamic shear modulus. Gd is the corresponding dynamic shear modulus.for *γ*_*d*_.

From Eq (2), we can obtain:

$$\frac{1}{G_{d}}=\frac{\tau_{d}}{\gamma_{d}}=a+b\gamma_{d}$$ (4)

By performing regression analysis, we can obtain the relationship curve between shear modulus ratio and strain.

To calculate the damping ratio of the soil, use the following formula:

$$\lambda =\lambda_{\max}{\left(1-\frac{G_{d}}{G_{d\max}}\right)}^{M}$$ (5)

In the formula, *λ* represents the damping ratio corresponding to G_d_, *λ*_*max*_ is the maximum damping ratio, *M* is the experimental parameter, *λ*_*max*_ and *M* is determined from the experimental data.

## Experimental results and analysis

### Dynamic shear modulus results and analysis

#### The influence of confining pressure on dynamic shear modulus and damping ratio of seismically poor sand soil

The dynamic shear modulus of a soil refers to the ratio of shear stress to shear strain under dynamic loading conditions. It is an important parameter that characterizes the dynamic behavior of the soil.

**Fig 4:**
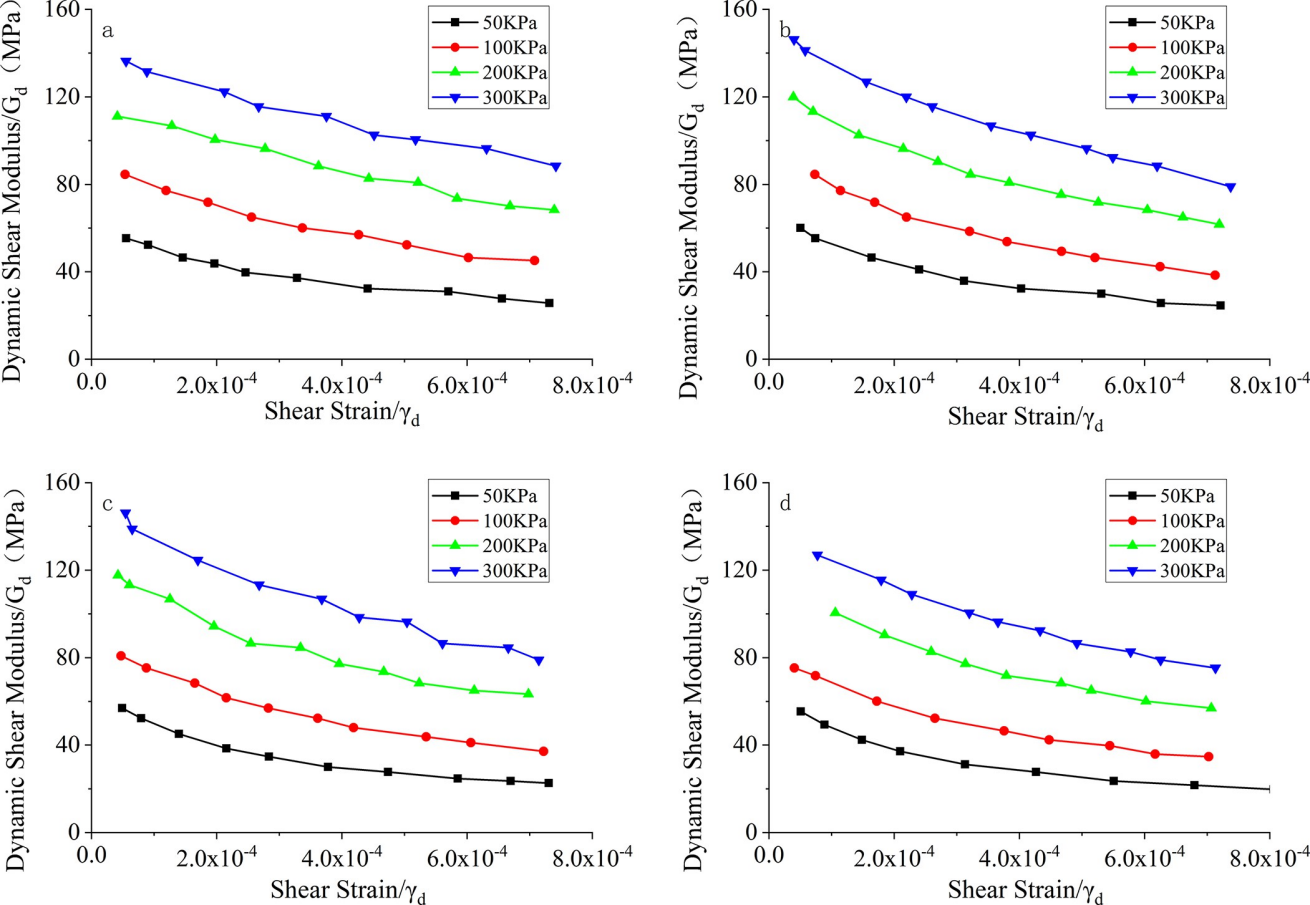
The comparative curves of G_d_-γ_d_ for seismic resistant poor sand soils under different confining pressures. a. Aw = 0; b. Aw = 2%; c. Aw = 4%; d. Aw = 8%.

The original data of the experiment can be found in S1 Table. Fig 4 presents the G_d_-γ_d_ curves of the seismic resistant poor sand soil under different confining pressures. It can be observed that the dynamic shear modulus of the samples decreases with increasing shear strain. The dynamic shear modulus gradually decreases with increasing shear strain. This is because the dynamic shear modulus is negatively correlated with the shear strain, as indicated by Eq (4). The reduction in dynamic shear modulus is more significant at lower shear strains, while it becomes less pronounced at higher shear strains. This pattern reflects the nonlinearity of the soil, which is because the mixture of sand and cement introduces a certain degree of structural integrity to the specimen. Especially at higher shear strains, this structural integrity helps resist the reduction of the dynamic shear modulus. Furthermore, with a constant cement content, the dynamic shear modulus increases with increasing confining pressure. The magnitude of the increase in the dynamic shear modulus is relatively uniform, and the connecting lines of the data points form a hyperbolic shape, which is consistent with previous understanding.

**Fig 5:**
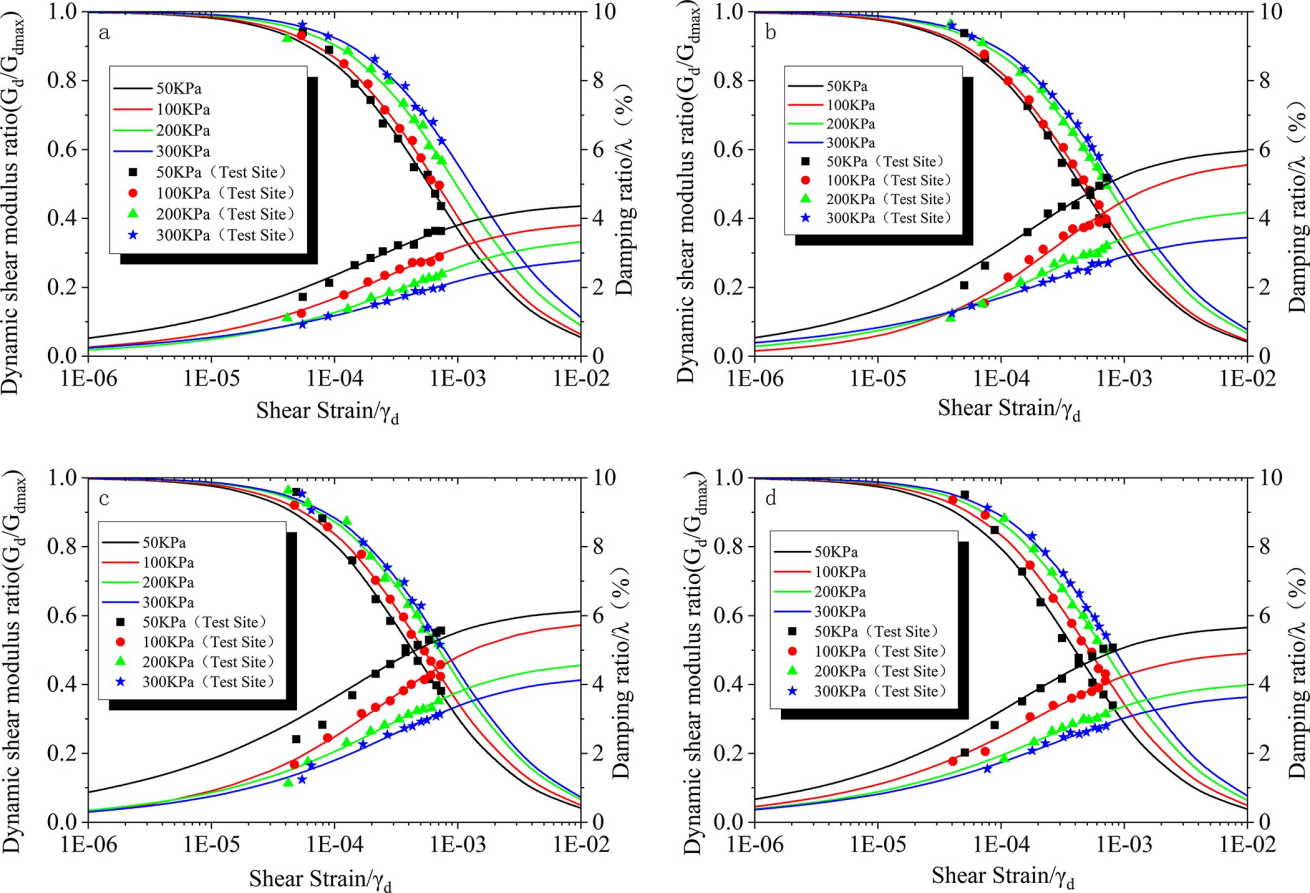
Relationship curves of G_d_/G_dmax_-γ_d_ and λ-γ_d_ for seismic resistant poor sand soils under different confining pressures. a. Aw = 0; b. Aw = 2%; c. Aw = 4%; d. Aw = 8%.

Fig 5 shows the relationship curves of G_d_/G_dmax_-γ_d_ and λ-γ_d_ for seismic-resistant poor sand soils under different confining pressures. The maximum dynamic shear modulus of the specimens is normalized to obtain the experimental points in the figure. When the experimental points are matched, the dynamic shear modulus ratio curve is obtained. From Fig 5, it can be observed that the dynamic shear modulus ratio decreases with increasing shear strain. At the same strain level, the dynamic shear modulus ratio increases with increasing confining pressure, which is consistent with the pattern of variation of the dynamic shear modulus.

The damping ratio of the specimens increases with increasing shear strain. At the same shear strain level, the damping ratio decreases with increasing confining pressure. As the confining pressure increases, the specimen experiences higher forces and exhibits better overall integrity, leading to a decrease in the damping ratio. When the experimental points with smooth curves are connected, it can be observed that as the confining pressure increases, the distance between adjacent curves becomes smaller, indicating a decreasing rate of the damping ratio.

#### The cement content has an impact on the dynamic shear modulus and damping ratio of seismic resistant poor sand soils

**Fig 6:**
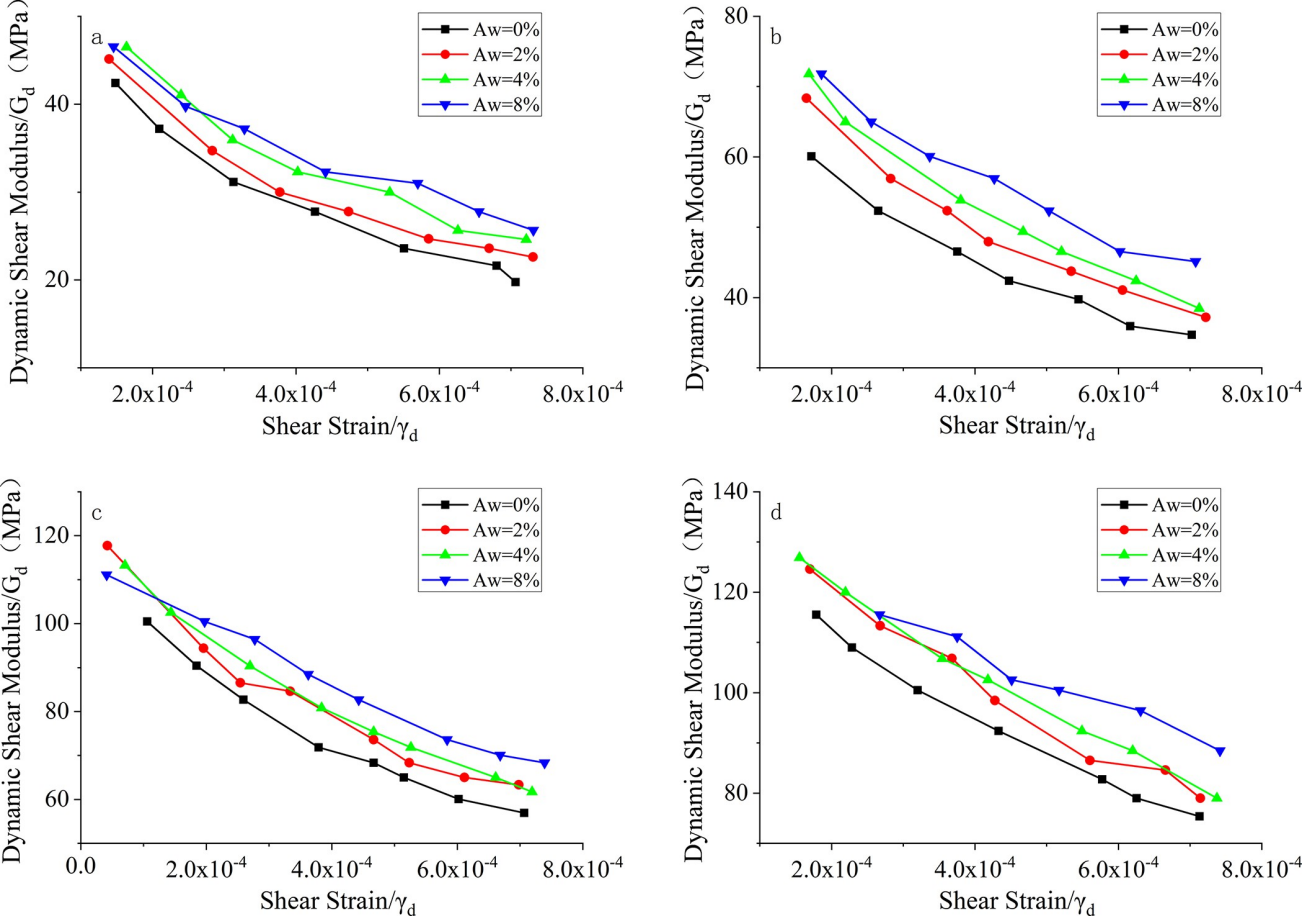
G_d_-γ_d_ curves of seismic poor sand with different cement contents. a. σ = 50kPa; b. σ = 100kPa; c. σ = 200kPa; d. σ = 300kPa.

Fig 6 shows the relationship between the dynamic shear modulus (G_d_) and the shear strain (γ_d_) of seismically poor sand with different cement contents. From Fig 6, it can be observed that the dynamic shear modulus of the soil increases significantly when cement is added compared to pure sand (0% cement content). The dynamic shear modulus of the samples increases with increasing cement content. Cement acts as a binder in the samples, promoting the cohesion of the sand particles, enhancing the overall integrity, and increasing the stiffness of the samples, resulting in an increase in the dynamic shear modulus. At lower strain levels, the difference in dynamic shear modulus between samples with different cement content is relatively small. However, under higher strain conditions, the dynamic shear modulus increases rapidly with increasing cement content. For example, in Fig 6(D) at a confining pressure of 300 kPa, when the strain is 3E-4, the dynamic shear modulus of pure sand is 102.5 MPa, while the dynamic shear modulus of the sample with 8% cement content is 113 MPa, indicating an increase of 10.24%. Similarly, at a strain of 7E-4, the dynamic shear modulus of pure sand is 76 MPa, while the dynamic shear modulus of the sample with 8% cement content is 91 MPa, indicating an increase of 19.74%. It can be concluded that the cement content has a significant influence on the dynamic shear modulus of seismicly poor sand."

**Fig 7:**
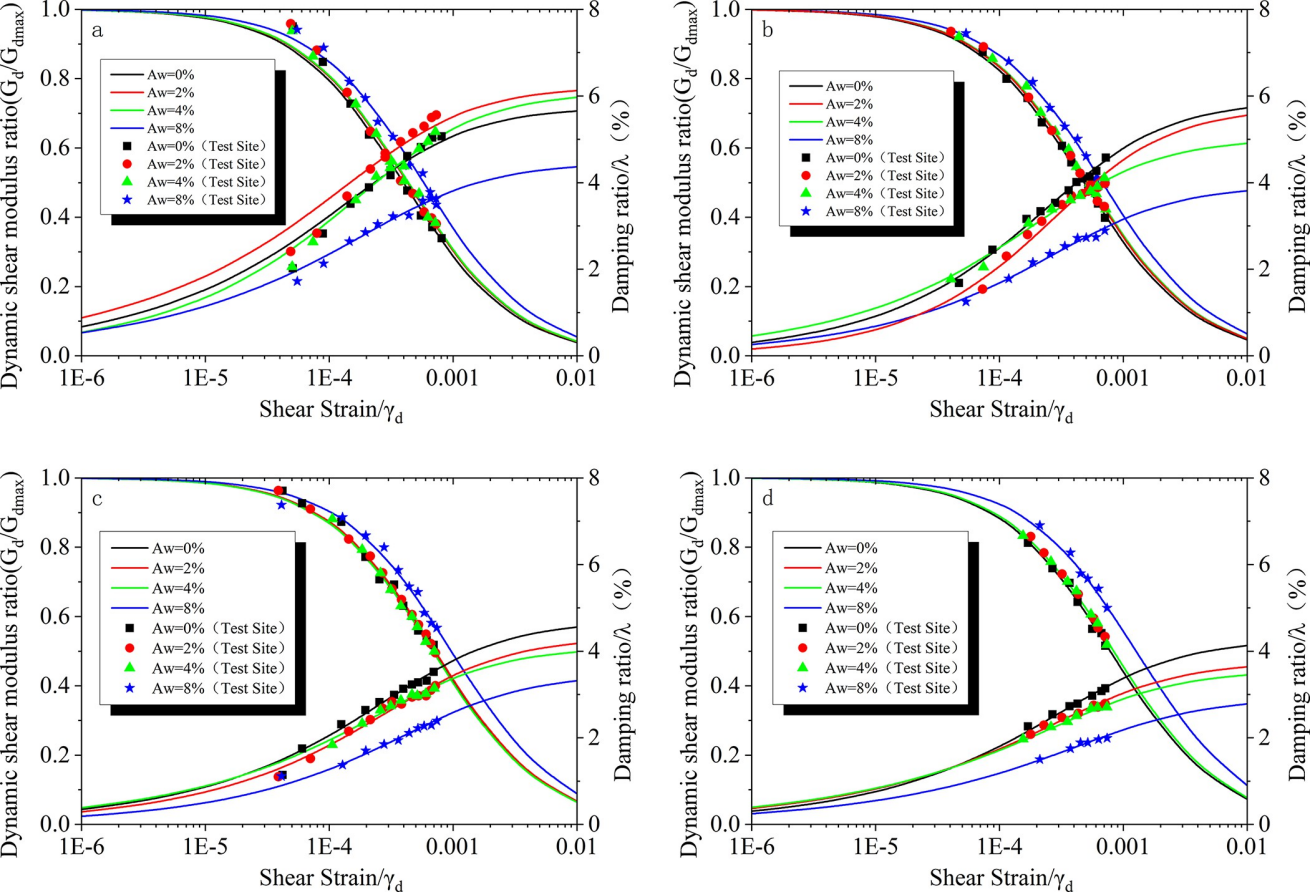
Shows the G_d_/G_dmax_-γ_d_ and λ-γ_d_ relationship curves of seismicly poor sand with different cement contents. a. σ = 50kPa; b. σ = 100kPa; c. σ = 200kPa; d. σ = 300kPa.

Fig 7 represents the G_d_/G_dmax_-γ_d_ and λ-γ_d_ relationship curves of seismic poor sand with different cement contents. From Fig 7, it can be observed that the dynamic shear modulus ratio of the samples decreases with increasing shear strain. Under different cement contents, the dynamic shear modulus ratio of the samples is significantly higher than that of the pure sand sample with 0% cement content, with the most pronounced effect observed at a cement content of 8%. When the shear strain is constant, the dynamic shear modulus ratio increases with increasing cement content, following the same trend as the dynamic shear modulus. The damping ratio of the samples increases with increasing shear strain, and when the shear strain is constant, the damping ratio decreases with increasing cement content. This is because the cement acts as a binder, increasing the contact points between the sand particles and improving the overall integrity and stiffness of the samples, resulting in a reduced energy dissipation and a lower damping ratio. Furthermore, it can be seen from the graph that the difference in damping ratio between the samples with 0%, 2%, and 4% cement content is minimal, while the samples with 8% cement content exhibit a lower damping ratio, indicating a more significant reduction. Based on the experimental data, the average difference in damping ratio between samples with 0% and 2% cement content is 0.12, between samples with 2% and 4% cement content is 0.18, and between samples with 4% and 8% cement content is 1.33. This indicates that the cement content has an influence on both the dynamic shear modulus and the damping ratio of the soil."

#### The maximum dynamic shear modulus of poor seismic sand

The maximum dynamic shear modulus of seismic poor sand for each sample can be calculated using Formulas (2) and (3), and the results are shown in Table 3. From the table, it can be observed that, at a constant cement content, the maximum dynamic shear modulus increases with the increase in confining pressure, exhibiting a predominantly linear relationship. The confining pressure has a significant influence on the maximum dynamic shear modulus. As the confining pressure increases, the sample becomes stiffer with a larger stiffness, resulting in an increase in the maximum dynamic shear modulus.

**Table 3:** Maximum dynamic shear modulus of anti-seismic poor sand under various working conditions.

σ/kPa G_dmax_/MPa Aw/%	0	2	4	8
**50**	58.8375	64.0376	69.3472	78.2434
**100**	85.8425	89.4807	91.8297	98.5267
**200**	120.5308	124.5046	126.2591	133.9423
**300**	141.6644	152.2405	153.2644	159.0610

When the confining pressure remains constant, the maximum dynamic shear modulus increases with an increase in the cement content. With the increase in cement content and the effect of cementitious bonding, the specimen becomes "stiffer" with increased stiffness, resulting in an increase in the maximum dynamic shear modulus.

**Fig 8:**
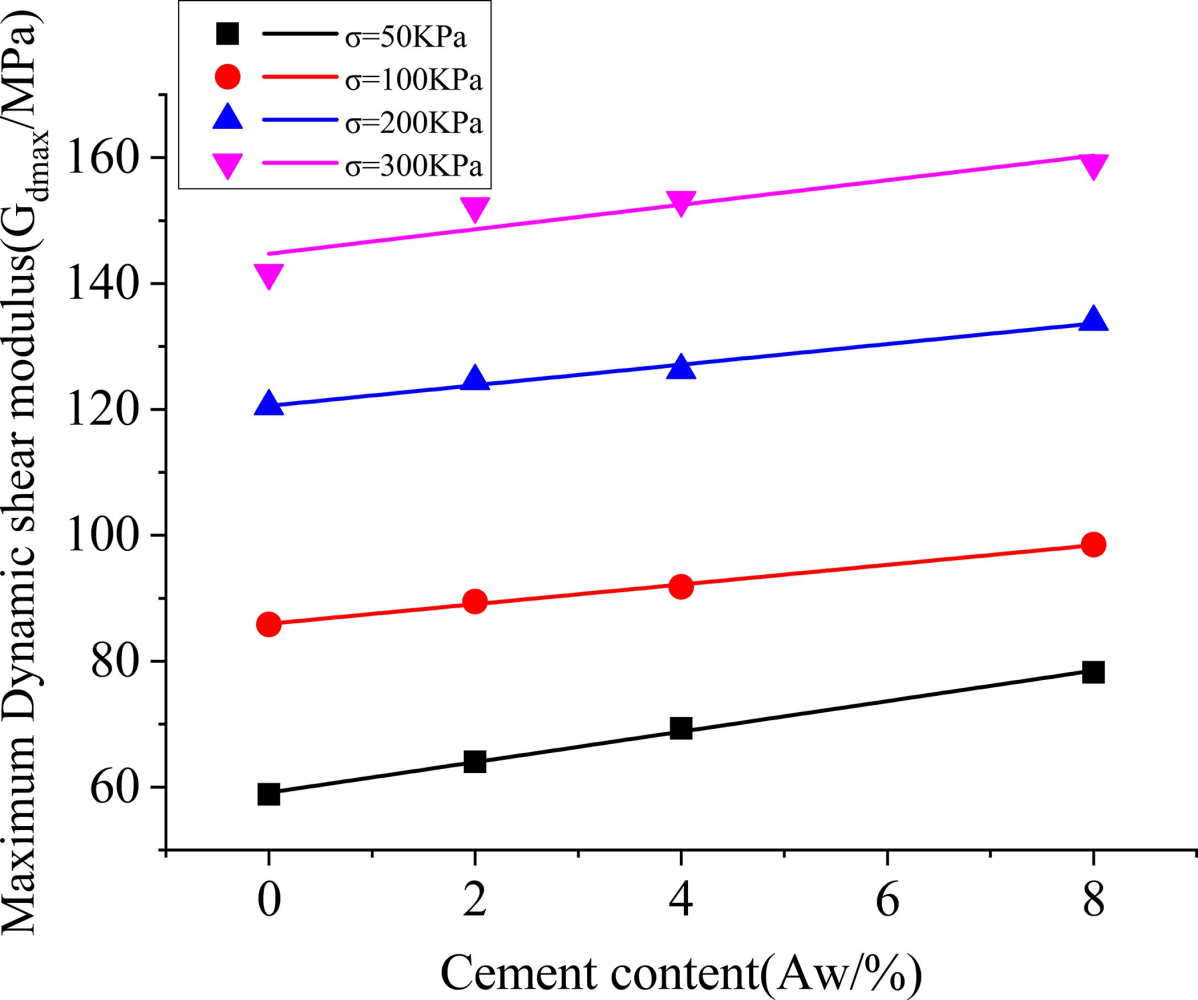
Relationship between the maximum dynamic shear modulus and the cement content and the confining pressure.

The trend of the variation in the maximum dynamic shear modulus can be observed more intuitively by linearly fitting the data points of the maximum dynamic shear modulus for different confining pressures and cement contents, as shown in Fig 8.

The expression for the linear fitting is as follows:

$$G_{d\max}=A+BA_{w}$$ (6)

In the equation, *Aw* represents the cement content (%), A represents the intercept, and B represents the slope. The values of A and B in the equation can be obtained from the linear regression, as shown in Table 4.

**Table 4:** Values of A and B in Eq (6).

parameter	Confining Pressure(kPa)			
	50	100	200	300
**A**	59.138	85.958	120.583	144.735
**B**	2.422	1.560	1.636	1.949

### Experimental results and analysis of comparative study between improved sand and remolded sand

#### Dynamic shear modulus and damping ratio

**Fig 9:**
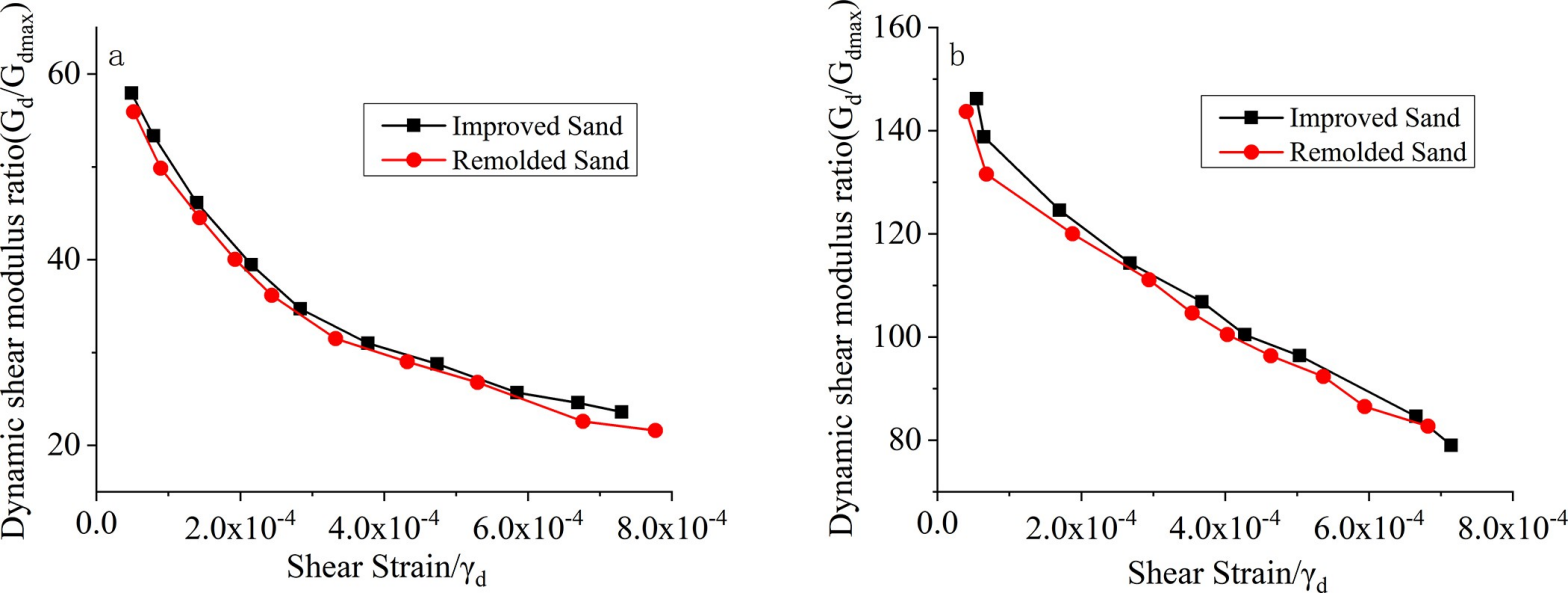
Relationship between the dynamic shear modulus and shear strain for both the modified sand and the reconstituted sand. a. σ = 50kPa; b. σ = 300kPa.

**Fig 10:**
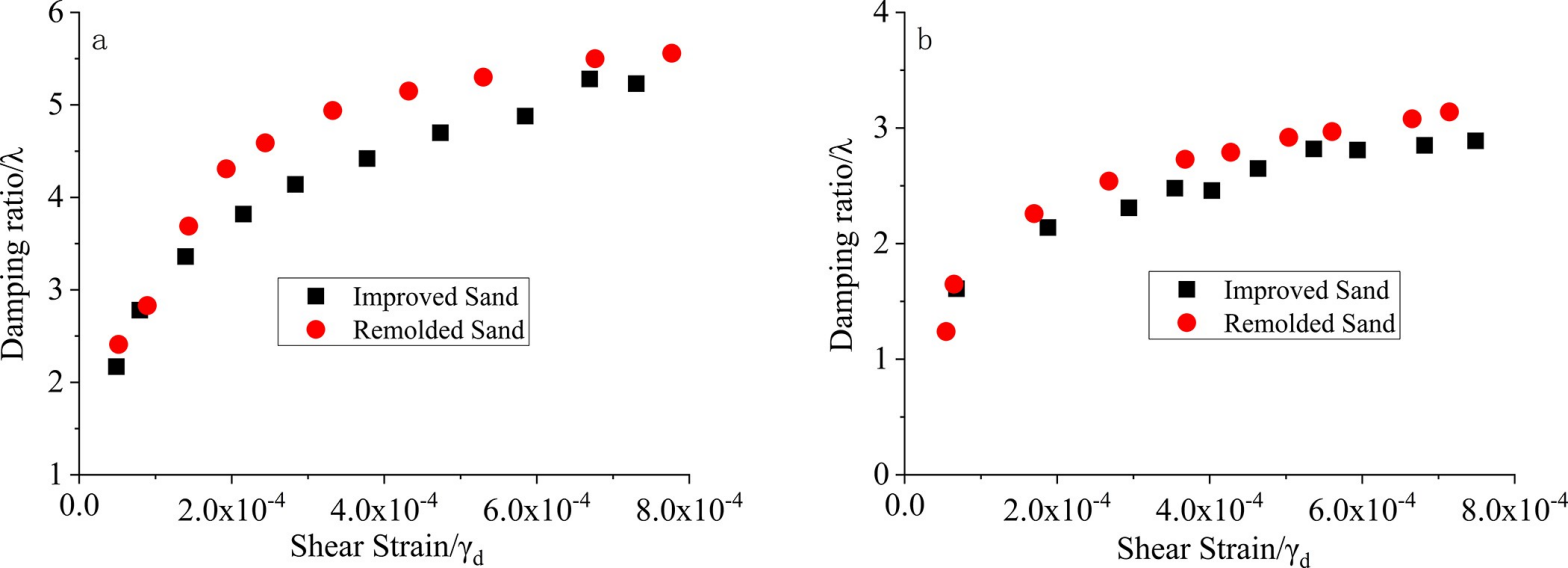
Scatter plot of the damping ratio variation with shear strain for improved sand and remolded sand. a. σ = 50kPa; b. σ = 300kPa.

According to the experimental plan, the crushed modified sand under the third set of conditions was subjected to reconstitution tests. Fig 9 shows the comparison of the variation of the dynamic shear modulus with the shear strain for modified sand and reconstituted sand under confining pressures of 50 kPa and 300 kPa. Fig 10 presents the scatter plot of damping ratio versus shear strain for modified sand and reconstituted sand under confining pressures of 50 kPa and 300 kPa. From Figs 9 and 10, it can be observed that there are significant differences between reconstituted sand and modified sand in terms of both the dynamic shear modulus and the damping ratio. The dynamic shear modulus of the reconstituted sand is generally smaller than that of modified sand, whereas the damping ratio of reconstituted sand is greater than that of modified sand. This trend holds for both 100 kPa and 200 kPa confining pressures. This can be attributed to the degradation or loss of the original cementation effect in the reconstituted sand after reconstitution, leading to a decrease in the dynamic shear modulus and an increase in the damping ratio. It is evident that the cement content has a significant effect on the improvement of seismic performance in poor sandy soils.

#### Maximum dynamic shear modulus

The numerical values of the maximum dynamic shear modulus for the improved sand and the corresponding remolded soil can be found in Table 5. Table 6 provides the recommended values for the increase in magnitude of the maximum dynamic shear modulus considering the improvement effect of the sand. It can be observed that, under a constant confining pressure, the maximum dynamic shear modulus of the remolded soil is smaller than that of the improved sand. Based on the statistical results, the range of reduction in the maximum dynamic shear modulus for the remolded soil compared to the improved sand is 3.6 MPa to 12.6 MPa, with a decrease magnitude ranging from 3% to 23%.

**Table 5:** Values of maximum dynamic shear modulus for improved sand and respective remolded soil.

Parameters	Confining pressure(kPa)			
	50	100	200	300
**Improved sand**	69.347	91.829	126.259	153.264
**Remolded sand**	59.679	86.717	122.627	146.460

**Table 6:** Recommended values for the increase in maximum dynamic shear modulus considering the improvement effect of the soil(%).

Confining pressure(kPa)	50	100	200	300
**Recommended values for the increase in magnitude(%)**	16.2000	5.8950	2.9618	4.6456

#### Recommended values for the variation range of the dynamic shear modulus ratio and damping ratio, considering the improvement effect on seismic-poor sandy soil

Based on comprehensive experimental results and analysis, the influence of cement content on the dynamic characteristics of seismicly poor soil can be determined. When cement is used to improve the poor seismic soil, the dynamic shear modulus ratio and dynamic shear modulus of the soil are higher compared to the unimproved soil, while the damping ratio is lower. Based on this, in order to quantify the effect of different cement content on the improvement of seismic poor soil, recommended values that reflect the changes in the soil’s dynamic shear modulus ratio and damping ratio after improvement are provided through calculations. These values can be obtained by multiplying appropriate coefficients with the data obtained from the experiments, and the coefficient values can be referenced from Table 7. The recommended values can serve as a reference for seismic design, subgrade treatment, and engineering construction in practical projects.

**Table 7:** Recommended values for the increase in dynamic shear modulus ratio and the decrease in damping ratio considering cement content improvement in seismic poor soil(%).

Confined Pressure(kPa)	Parameters	Shear strain/γ_d_(10^−4^)							
		0.05	0.1	0.5	1	5	10	50	100
**50**	G_d_/G_dmax_	0.06	0.12	0.57	1.04	3.02	3.97	5.29	5.52
	λ	-18.13	-16.32	-12.03	-10.20	-6.51	-5.42	-4.17	-3.97
**100**	G_d_/G_dmax_	0.04	0.08	0.39	0.72	2.23	3.03	4.24	4.45
	λ	-15.96	-8.23	-2.98	-3.09	-8.20	-9.57	-10.93	-11.12
**200**	G_d_/G_dmax_	0.01	0.02	0.07	0.13	0.46	0.66	0.98	1.04
	λ	-12.15	-8.03	-4.06	-6.83	-7.30	-8.08	-8.95	-9.08
**300**	G_d_/G_dmax_	0.07	0.14	0.67	1.27	4.54	6.67	10.68	11.55
	λ	-36.13	-29.85	-13.25	-5.51	-9.91	-13.84	-18.03	-18.65

## Conclusions

Based on the resonant column tests conducted on four different cement contents of seismic poor soil, the following conclusions can be drawn regarding the variation of dynamic shear modulus and damping ratio under four different confining pressures and the effect of improving the dynamic characteristics parameters of seismic poor soil: Cement has a significant positive effect on the dynamic characteristics of anti-seismic poor sandy soil. The effect of improvement varies with different cement content, and the most significant improvement is observed at a cement content of 8%. With the increase in cement content and confining pressure, both the dynamic shear modulus and the dynamic shear modulus ratio increase, while the damping ratio decreases. As the confining pressure increases, the decrease in damping ratio is uneven, but with the increase in cement content, the reduction in damping ratio is more pronounced. This has a positive impact on the seismic safety assessment. A relationship equation between the maximum dynamic shear modulus and the cement content for improved anti-seismic poor sandy soil with cement content less than 8% has been established. Recommended values have been provided for the change in the dynamic shear modulus ratio and the damping ratio, considering the improvement effect of anti-seismic poor sandy soil. These results serve as a reference for foundation treatment in sandy soil engineering sites. It is important to note that due to limitations in the experimental apparatus, the consolidation ratio for all experiments in this study was set to 1. However, in practical situations, most soil conditions do not align with this, and further research is needed to explore the effects of the consolidation ratio and related compaction density.

## Supporting information

S1 Table The raw data obtained from the experiment. (XLSX)
